# Editorial: Properties and Applications of Ionic Liquids in Energy and Environmental Science

**DOI:** 10.3389/fchem.2020.627213

**Published:** 2020-12-15

**Authors:** Faiz Ullah Shah, Rong An, Nawshad Muhammad

**Affiliations:** ^1^Chemistry of Interfaces, Luleå University of Technology, Luleå, Sweden; ^2^Department of Materials Science and Engineering, Herbert Gleiter Institute of Nanoscience, Nanjing University of Science and Technology, Nanjing, China; ^3^Interdisciplinary Research Centre in Biomedical Materials (IRCBM) COMSATS University Islamabad, Islamabad, Pakistan

**Keywords:** ionic liquids, energy, biomass, lubrication, physical properties

Ionic liquids (ILs) are salts comprising cations and anions, and usually liquids at or below 100°C. The salts that are liquids at room temperature are generally called as room-temperature ionic liquids (RTILs) (Seddon, 1999; Welton, 2018; Singh and Savoy, 2020). In contrast to inorganic salts, ILs have much lower melting points primarily due to the larger sizes of either the cations or the anion, or both. In addition, their molecular structures possess a high degree of asymmetry which affects the ionic packing and thus decrease the Coulombic attraction between the ions (Welton, 1999). The first IL was prepared by Walden (1914) through a neutralization of ethylamine with a concentrated nitric acid. The resulting ethylammonium nitrate IL [${\text{EtNH}}_{3}^{+}$][${\text{NO}}_{3}^{-}$] revealed a melting point of 12°C (Walden, 1914). At that time, ILs did not receive any significant attentions of the scientific community but later on during the 1980s, ILs appeared to be promising in various electrochemical applications (Wilkes et al., 1982; Fannin et al., 1984).

Unlike molecular liquids, ILs exhibit many unique properties making them promising solvents for various industrial applications. Some of the physicochemical properties of ILs include high polarity, negligible volatility, high thermal stability, high ionic conductivity, low melting point, and structural designability (Shah et al., 2013). The latter can be exploited in tuning the physicochemical properties of ILs and making them task specific for challenging applications where molecular liquids cannot be used (Plechkova and Seddon, 2008). Over the past two decades, ILs have been considered as promising solvents with unique abilities in organic synthesis, catalysis, and electrochemistry, separation of metals, gas separation, biomass processing, pharmaceuticals, tribology and energy storage devices such as batteries, supercapacitors, fuel cells, etc. (MacFarlane et al., 2014; Bhattacharyya et al., 2016, 2017; Egorova et al., 2017; Shah et al., 2017, 2020; Clarke et al., 2018; Khan et al., 2018; An et al., 2019; Mezzetta et al., 2019; Khan and Shah, 2020; Nie et al., 2020; Spange et al., 2020).

Deep eutectic solvents (DESs) are emerging as a new class of solvents having many comparable properties with ILs, although these two systems are very different from each other. In contrast to ILs that are composed of cations and anions, DESs are made by mixing Lewis or Brønsted acids and bases, which may contain anionic and/or cationic species (Smith et al., 2014). Compared to ILs, which are extensively studied over the past few decades, the number of publications on DES are very limited. However, a great interest has been recently seen in DES as environmentally benign alternatives for various applications such as synthesis, gas adsorption, biomass processing, electrolytes for energy storage devices, and metal processing applications.

Hua and Shi have proposed a strategy that uses a non-corrosive green lubricant with dissolved lignin in ILs (lignin-[Choline][L-Proline]) to physically adsorb onto steel-DLC contacts. This green IL lubricant exhibits a significant improvement in wear resistance as compared with the commercially available lubricants. Tong et al. have provided an essential molecular insight into understanding the effect of concentration of lithium salt on IL electrolytes using combined experimental and molecular dynamics simulation. The analysis of physicochemical properties, transport properties, and coordination structures of various IL electrolytes in different concentration of lithium salt shed light on the change in structural and physical properties at a micro-scale level. Lethesh et al. have prepared some hydroxyl-containing pyridinium based ILs with different alkyl side chain on cation with Br^−^ and [Tf2N]^−^ anions. The different alkyl side chains strongly affected the physiochemical and electrochemical properties of these ILs. The experimental physicochemical properties are in line with the predicted values. The electrochemical window was measured in the range of 3.0–5.4 V. The DFT/COSMO-RS based calculations for the 3-methyl/ethyl ILs with 3Tf_2_N and 10Tf_2_N showed the highest conductivity values (0.366–0.383 κ/S m^−1^) among the synthesized ILs.

Liu et al. have acquired the data of CO_2_ solubilities and Henry's constant in Deep Eutectic Solvents (DES) from literature and processed through COSMO-RS-based calculations for improved verification and screening ability. The corrected COSMO-RS with adjustable universal parameters has great reliability to predict the CO_2_ solubility in DESs and the ARDs for the logarithmic CO_2_ solubility were 6.8, 5.2, 6.6, and 4.7%, in the DESs of different HBA and HBD ratios. Naz et al. have suggested IL-based catalysts containing [BMPy]Cl coupled with a number of different metal chlorides for effective deconstruction of wheat straw biomass. The IL-metal catalytic systems showed up to 86% recovery for [BMPy]^+^${\text{CoCl}}_{3}^{-}$ and outstanding recycling abilities. These IL-based catalytic systems were proposed as sustainable solutions for conversion of wheat straw to valuable products.

The Research Topic “Properties and Applications of Ionic Liquids in Energy and Environmental Science” presents research articles that will provide valuable feedback to chemists for designing efficient ILs for various applications in energy and environmental applications.

## Author Contributions

All authors listed have made a substantial, direct and intellectual contribution to the work, and approved it for publication.

## Conflict of Interest

The authors declare that the research was conducted in the absence of any commercial or financial relationships that could be construed as a potential conflict of interest.
